# Optoelectronic and Electrochemical Properties of Vanadium Pentoxide Nanowires Synthesized by Vapor-Solid Process

**DOI:** 10.3390/nano6080140

**Published:** 2016-07-29

**Authors:** Ko-Ying Pan, Da-Hua Wei

**Affiliations:** Institute of Manufacturing Technology and Department of Mechanical Engineering, National Taipei University of Technology (TAIPEI TECH), Taipei 10608, Taiwan; koyingpan@mail.ntut.edu.tw

**Keywords:** V_2_O_5_ nanowires, VS method, spiral growth mechanism, screw dislocation, PL spectrum, four-point probe method, electrical resistivity, cyclic voltammetric curve

## Abstract

Substantial synthetic vanadium pentoxide (V_2_O_5_) nanowires were successfully produced by a vapor-solid (VS) method of thermal evaporation without using precursors as nucleation sites for single crystalline V_2_O_5_ nanowires with a (110) growth plane. The micromorphology and microstructure of V_2_O_5_ nanowires were analyzed by scanning electron microscope (SEM), energy-dispersive X-ray spectroscope (EDS), transmission electron microscope (TEM) and X-ray diffraction (XRD). The spiral growth mechanism of V_2_O_5_ nanowires in the VS process is proved by a TEM image. The photo-luminescence (PL) spectrum of V_2_O_5_ nanowires shows intrinsic (410 nm and 560 nm) and defect-related (710 nm) emissions, which are ascribable to the bound of inter-band transitions (V 3d conduction band to O 2p valence band). The electrical resistivity could be evaluated as 64.62 Ω·cm via four-point probe method. The potential differences between oxidation peak and reduction peak are 0.861 V and 0.470 V for the first and 10th cycle, respectively.

## 1. Introduction

Vanadium pentoxide, V_2_O_5_, has been fully exploited in electrochemical energy storage, catalysis and gasochromic coloration [1,2,3,4,5,6,7]. In general, V_2_O_5_ nanostructures are highly regarded by researchers due to their redox-activity and layered structures, which provide the inserts for electrochemical intercalation/deintercalation of lithium ions [8,9]. Some published literatures disclose the practical applications of V_2_O_5_ nanostructure, including electrochemical super-capacitors and energy storage gadgets [10,11,12]. Also, compared to several thin film materials, low-dimensional vanadium oxide nanostructures are functional as well as attractive as gas sensors of hydrogen [13,14]. In addition, coating platinum (Pt) as a catalyst on V_2_O_5_ nanostructures makes reactions with hydrogen easy, meaning that they could behave much more sensitively toward the leakage of hydrogen than uncoated V_2_O_5_ nanostructures. It should be noted that many technical methods, namely hydrothermal synthesis [15,16], sol-gel process [17,18] and evaporation-condensation growth [19,20], have been rapidly expanded in the syntheses and formation of one-dimensional V_2_O_5_ nanostructured materials; however, very little of the previous research about fabricating V_2_O_5_ nanowires concerns the VS method. The VS method belongs to a bottom-up process in fabricating nanostructured materials, which uses a gas atom or a gas molecular as the basis unit from which chemical vapor deposition (CVD) is developed. Generally, the gas atom or gas molecular of materials is directly deposited on the substrate, which is quite different from vapor-liquid-solid (VLS) and solid-liquid-solid (SLS) approaches. Both VLS and SLS approaches need catalysts as the precursors, which act as an intermedium in the adsorption of growth species. In other words, the catalysts in the VLS and SLS methods are the nucleation sites leading to the growth of nanostructured materials. In terms of the VS method, Zhao et al. [21] synthesized large-scale GaN nanorods via this method. The appearance of the GaN nanorods is cone-tip and the GaN nanorods might be grown along the screw dislocations, so the GaN nanorods belong to a spiral growth mechanism.

Yang et al. [22] adopted VS growth to employ magnesium oxide (MgO) nanowires on MgO substrates, and two noteworthy features of nucleation sites have thus been exhibited:

1. The MgO substrates are etched in 0.5 M NiCl_2_ solutions for 1 to 30 min to create nanoscale pits/projections, then the depositions of MgO nanowires have been grown on the nanoscale pits/projections of MgO (100) substrates by VS method. In other words, the nanoscale pits/projections are the nucleation sites of this VS method.
2. The MgO nanowires are directly grown on the MgO substrates without etching process. At the beginning of this reaction, the MgO nanoscale particles are deposited on the surface of MgO substrates, which include plenty of defects, i.e., steps, kinks and so on. These defects are the nucleation and the oriented growth of MgO nanowires.

In this task, a very simple and efficient fabrication, thermal evaporation, was presented to synthesize V_2_O_5_ nanowires via VS method. The samples were characterized by scanning electron microscopy (SEM), energy-dispersive X-ray spectrometer (EDS), X-ray diffraction (XRD) and transmission electron microscopy (TEM). Regarding the optoelectronic property, the luminescence and electronic property of the synthesized V_2_O_5_ nanowires were investigated by photo-luminescence (PL) spectrum and the four-point probe method, respectively. Furthermore, the performance of electrochemical examination was recorded by cyclic voltammetry (CV).

## 2. Experimental Method

**Figure 1:**
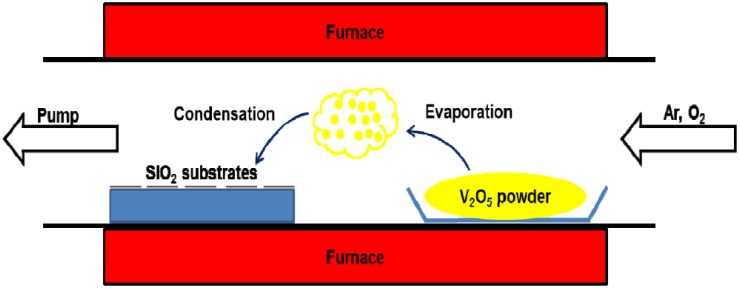
Schematic diagram illustrating the thermal evaporation set-up with three main processes including evaporation, reaction and condensation.

V_2_O_5_ nanowires were fabricated on silicon substrates, a commercial item with a growth plane of (100), by thermal evaporation. Good quality 0.5 g V_2_O_5_ powders (Alfa, purity 99.5%, 325 mesh, Heysham, Lancashire, UK) and several silicon substrates were situated in an aluminum oxide (Al_2_O_3_) boat, and then the Al_2_O_3_ boat was placed in the heating area of a quartz tube, as illustrated in Figure 1. These substrates employed in this experiment are commercial silicon wafers of (001). In advance, The Si substrates were prepared without chemical etchings or physical damage, but were cleaned ultrasonically in acetone. During the growth of V_2_O_5_ nanowires, the pressure inside the quartz tube was maintained at 0.5 Torr by a rotary pump. The working parameters of this process were as follows: (1) Increasing the working temperature to 800 °C with 26 °C/min was at a continual flow rate of 30 sccm Ar gas; (2) Maintaining the temperature at 800 °C for 1 h was at a constant flow rate of combined gas of 30 sccm Ar and 30 sccm O_2_; (3) Cooling to the room temperature was under a constant flow rate of 30 sccm of Ar gas.

The morphology, crystal structure and chemical composition of the V_2_O_5_ nanowires were analyzed by a field emission scanning electron microscope (FESEM, Zeiss, SIGMA Essential, Jena, Germany), a high-resolution transmission electron microscope (HRTEM, JEOL, JEM-2010, Tokyo, Japan), a MAC glancing incident X-ray spectrometer with an incident angle of 0.5° (PANalytical, Almelo, The Netherlands), and energy-dispersive X-ray spectroscopy (EDS, Bruker, Hanau, Germany), respectively. Regarding the optical property of V_2_O_5_ nanowires, the measurement via PL (RENISHAW inVia, Wotton-under-Edge, UK) at room temperature under an irradiation (λ = 325 nm, 20 W/m^2^) of wavelength from 300 to 800 nm was undertaken. As for the electronic property of single V_2_O_5_ nanowire, the sample with regard to the electronic measurement was prepared by dual-beam focused ion beam system (FIB, FEI Helios Nanolab 600i System, Hillsboro, OR, USA), then the I-V curve was characterized between −3 V and 3 V by Keithley 4200 (Solon, OH, USA).

With respect to inquiring into the electrochemical properties of V_2_O_5_ nanowires, the cyclic voltammetric curve was plotted by an Electrochemical Workstation (CHI 627E, Austin, TX, USA) to investigate the potential of oxidation peak and reduction peak. The test cell includes three kinds of electrodes and one electrolyte in a beaker. A saturated calomel electrode (SCE) and a platinum foil were adopted as the reference electrode and counter electrode, separately. The working electrode was fabricated by mixing 80 wt. % V_2_O_5_ nanowires, 10 wt. % polyvinylidene difluoride (PVdF) and 10 wt. % carbon black in N-methyl pyrrolidinone (NMP) solvent to become a slurry. The slurry was spread onto an Al_2_O_3_ foil and evenly distributed on the Al_2_O_3_ foil, then the sample was placed in the oven to transform slurry into a solid. A volume of 2 M KCl aqueous solution was prepared as the electrolyte in the beaker-style three-electrode test cell. The cyclic voltammetry (CV) was conducted in a voltage range between −2 and 2 V at 50 m·VS^−1^ scan rate at room temperature.

## 3. Results and Discussion

**Figure 2:**
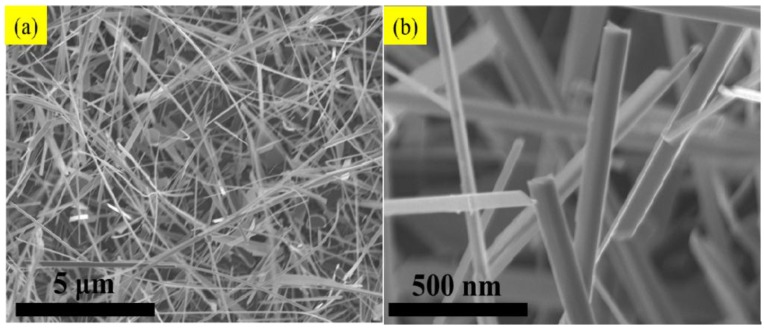
(**a**) Scanning electron microscope (SEM) image of V_2_O_5_ nanowires; (**b**) Enlarged SEM image of V_2_O_5_ nanowires.

**Figure 3:**
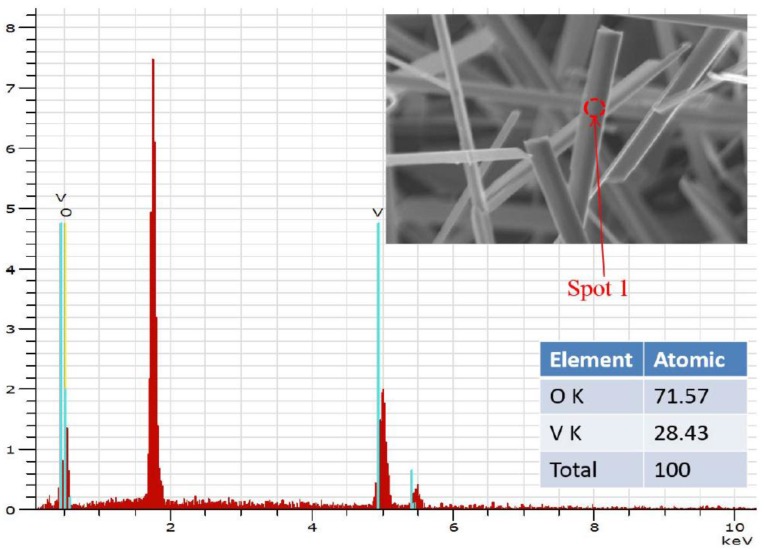
Energy-dispersive X-ray spectrometer (EDS) analysis at spot 1 of V_2_O_5_ nanowires. Inset: SEM image of spot 1.

**Figure 4:**
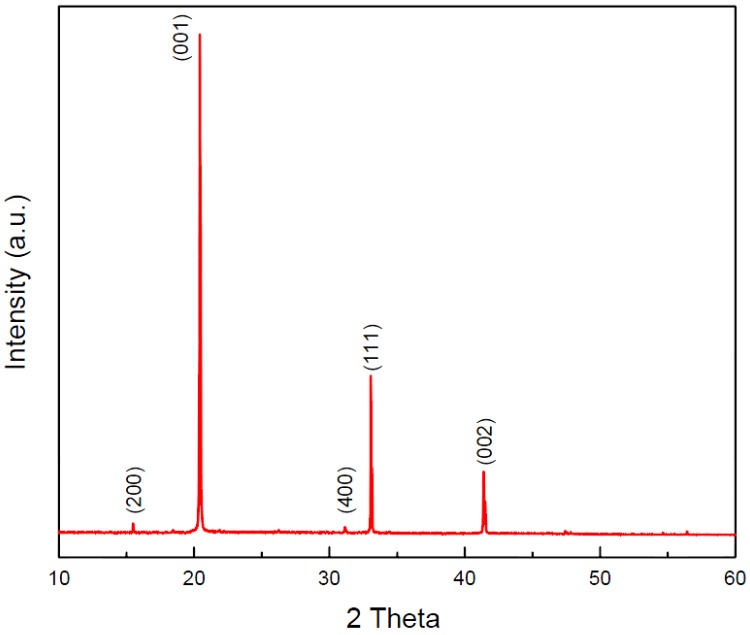
X-ray diffraction (XRD) pattern of V_2_O_5_ nanowires.

**Figure 5:**
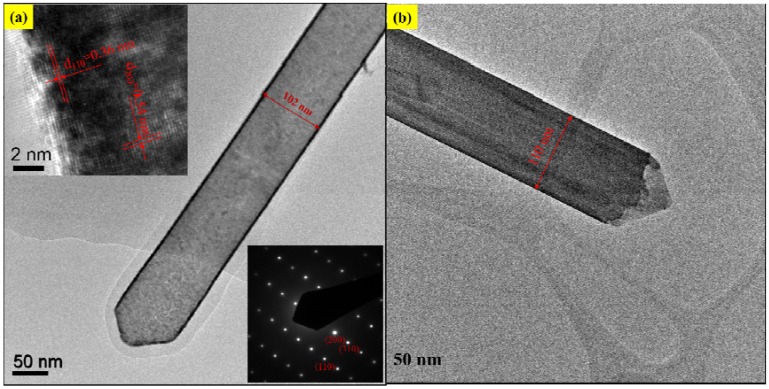
(**a**,**b**)Transmission electron microscope (TEM) images of a V_2_O_5_ nanowire. Upper left inset of (**a**): high-resolution transmission electron microscope (HRTEM) image. Lower right inset of (**a**): selected-area-electron-diffraction (SAED) pattern.

Figure 2 shows the SEM image of abundant V_2_O_5_ nanowires with diameter about 100 nm and length more than 10 μm, which have been prepared by the VS process. The micromorphology and microstructure of one single V_2_O_5_ nanowire were further investigated by observation of TEM. Figure 3, the EDS of spot 1 on the surface indicates the co-existence of V and O, and the percentages of V and O of an atom are 28.43 and 71.57, which is approaching to the ratio of V_2_O_5_. Herein, the above examinations totally testify to fabricate V_2_O_5_ nanowires effectively via this VS process. The XRD pattern of V_2_O_5_ nanowires is illustrated in Figure 4 exhibiting peaks of 2θ angles at 15.4°, 20.3°, 33.3°, and 41.3° corresponding to the reflection of (200), (001), (111) and (002) crystalline planes in the orthorhombic structure of V_2_O_5_, which makes a comparison of JCPDS: 77–2418, signifying that its structure belongs to P*mmn* and lattice parameters a, b and c are 11.51 Å, 3.564 Å and 4.368 Å, respectively. By calculating with Scherrer equation: *d = 0.9* λ*/B cos*θ (where *d* is grain diameter, λ is wavelength of the X-ray = 0.1541 nm (Cu-Kα), *B* is full width at half max of specific peak), the grain size is about 98.9 nm of (001). To observe the surfaces of V_2_O_5_ nanowires exactly, the low-magnification TEM image of a sample was taken in Figure 5a,b, and we demonstrated that the diameters of V_2_O_5_ nanowire are 102 and 110 nm, respectively. The upper left inset is HRTEM image of a V_2_O_5_ nanowire and the lower right inset is the selected-area-electron-diffraction (SAED) pattern of a V_2_O_5_ nanowire. After confirmation, one-dimensional V_2_O_5_ nanowires grow along the (110) plane with the interplanar spacing of 0.36 nm, which corresponds to the (110) plane of V_2_O_5_. Judging from the tip of a single V_2_O_5_ nanowire in Figure 5, the cone-tip is observed to be a proof of spiral growth mechanism [21,22,23]. As mentioned in the introduction and experimental method sections, the substrates were prepared without any preparations, for example, sputtering precursors, chemical and physical etchings processes. Therefore, the formation of V_2_O_5_ nanowires through the VS process is due to anisotropic growth and its growth mechanism is the screw dislocation in the crystal plane of (110).

**Figure 6:**
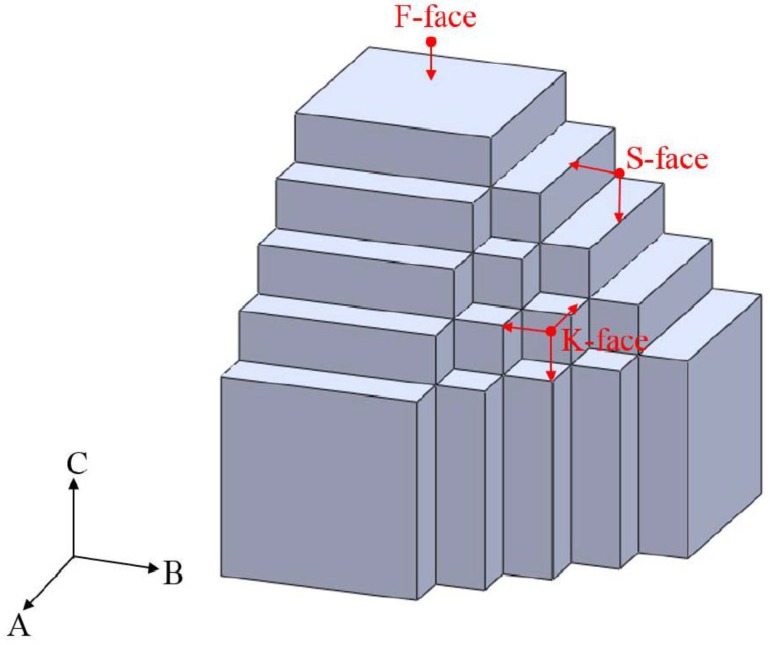
Schematic diagram illustrating the periodic bond chain (PBC) theory. Flat surfaces (F-face) with one PBC penetrate {100} planes. Stepped surfaces (S-face), {110} planes, own two PBCs. Kinked surfaces (K-face), {111} planes, have three PBCs.

Herein, the crystal growth via using VS process could be explained by Burton, Cabrera and Frank (BCF) theory [24]. In 1951, three outstanding scientists, Burton, Cabrera and Frank, promulgated a well-known paper “The growth of crystals and the equilibrium structure of their surface” [25], and a famous theory, BCF theory, was established to account for the growth of a crystal face in the presence of screw dislocations. In view of BCF theory [24,25], it should be pointed out that the existence of screw dislocations does not only ensure an advanced source of growth surface, but also achieves better growth rate. Various facets have a considerably different capability to accommodate dislocations, so the prominent role of dislocations on a certain facet can cause anisotropic growth for the formation of nanowires or nanorods. On the other hand, the periodic bond chain (PBC) theory provides a unique perspective in understanding the various growth rates and activities depending on different facets [26,27]. On the basis of PBC theory [26,27], the crystal surfaces are separated into three main groups: F (flat), S (stepped) and K (kinked) planes. In a simple cubic crystal (SCC), a VS process can be simply characterized as following the procedure illustrated in Figure 6. {100} faces belong to flat surfaces, which denotes F-face and they each have one PBC to penetrate one such plane. {110} faces are stepped surfaces, shortened to S-face, with two PBCs. {111} faces are kinked surfaces, abbreviated K-face, with three PBCs. For {110} surfaces, every single surface site is a step or ledge site; hence, any impinging atom would be incorporated wherever it absorbs. In terms of the S-face growth process, it always involves limited adsorption because the accommodation coefficient on {110} is unity, so all impinging atoms are obtained and incorporated into the growth surface. Therefore, in this study, the growth of V_2_O_5_ nanowires can be attributed to screw-dislocation-induced anisotropic growth, which is proven by both the TEM images in Figure 5a,b, and the above PBC theory.

**Figure 7:**
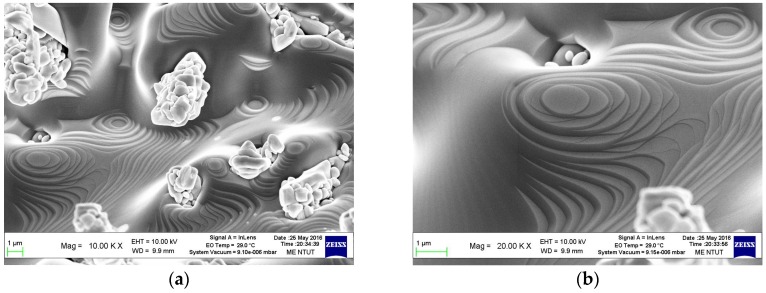
(**a**) SEM image taken from the sample prepared only at the beginning of the growth of V_2_O_5_ nanowires by vapor-solid (VS) process; (**b**) The enlarged SEM image.

Figure 7 shows the SEM images of the sample synthesized at the commencement of V growth species into V_2_O_5_ crystal nanostructures, which presents a step-line self-intersecting appearance, thus nucleating an island. Figure 7a,b is very similar to Figure 6, when the growth mechanism is the screw dislocation on the S-face in a SCC. Herein, this result provides clear proof to verify that the crystal growth of V_2_O_5_ nanowires is in accordance with the PBC theory. This theory denotes that the V_2_O_5_ nanowires grow along the {110} direction in VS process, so screw dislocations are required for the continued growth on a {110} surface that belongs to an S-face in PBC theory.

**Figure 8:**
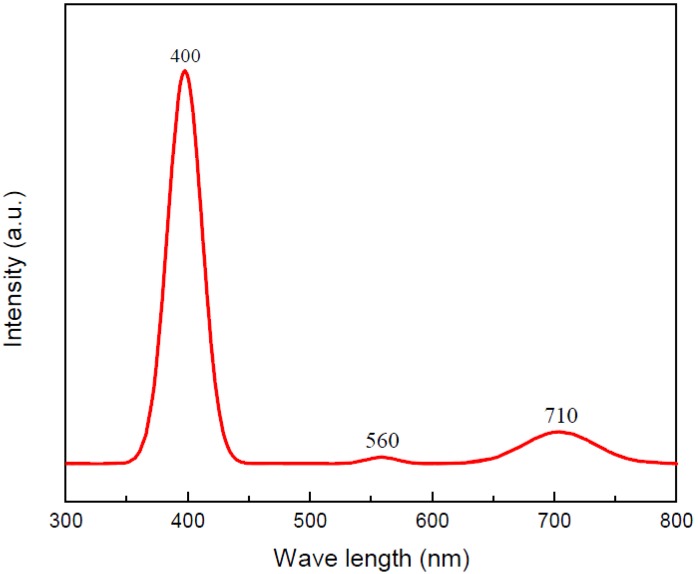
Photo-luminescence (PL) spectrum of V_2_O_5_ nanowires.

In order to evaluate the optical property, as depicted in Figure 8, the PL spectrum of V_2_O_5_ nanowires is recorded from 300 to 800 nm in the scale of wavelength at room temperature. Three luminescence peaks display the correspondence between the locations at 400, 560 and 710 nm and the photon energies approximated to 3.00, 2.21 and 1.75 eV, respectively. From the previous studies [19,28], the PL emission peaks located at 400 and 560 nm belong to intrinsic transition. Owing to the recombination of electron-hole pair from the bottom of the V 3d conduction band to the top of the O 2p valance band, the intensity of the PL spectrum is centered at 400 nm. The recombination of electron-hole pair from the V 3d split-off conduction band to the top of O 2p valence band causes the PL spectrum to peak at 560 nm. The light emission excited at 710 nm of PL spectrum belongs to extrinsic transition, which is derived from the recombination from the lowest defect donor band to the O 2p valence band. Generally, the defects are oxygen vacancies in V_2_O_5_ nanowires. According to the published papers [29,30], orthorhombic crystalline structure of V_2_O_5_ is composed of layers of VO_5_ square pyramids and these VO_5_ square pyramids regularly link at edges or corners. Additionally, the layers of V-O atoms connect the layers of O atoms in alternate layers along the c-axis. Because the bonding force is an electrostatic force in each layer, formations of oxygen vacancies are done spontaneously in the O layer between the nearest V-O layers in the (001) plane.

**Figure 9:**
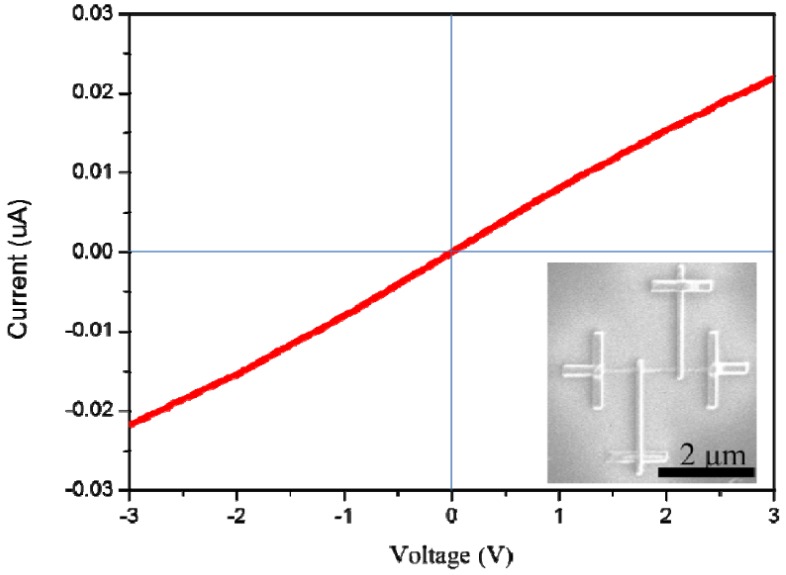
I-V curve (the red curve) of a single V_2_O_5_ nanowire measured by four-probe method. Lower right inset is the SEM image of the device fabricated by focused ion beam system (FIB).

Figure 9 demonstrates the measured current-voltage (I-V) curve of a single V_2_O_5_ nanowire, which has been measured by the four-point method. Under both the positive and negative bias of 3 V, the I-V characteristic curve shows an almost linear result and indicates a good Ohmic contact between the nanowire and four platinum electrodes. Moreover, the electrical resistivity (ρ) could be expressed as

(1) ρ=R×A/L

where R is the resistance of a nanowire; A is the cross section area of a nanowire; L is the length of a nanowire.

From the I-V characteristic curve, the resistance (R) equals 142 × 10^3^ kΩ. According to SEM and TEM images, L and A are equal to 2.3 μm and 102 nm^2^, respectively. The electrical resistivity could be calculated as 64.62 Ω·cm from Equation (1).

**Figure 10:**
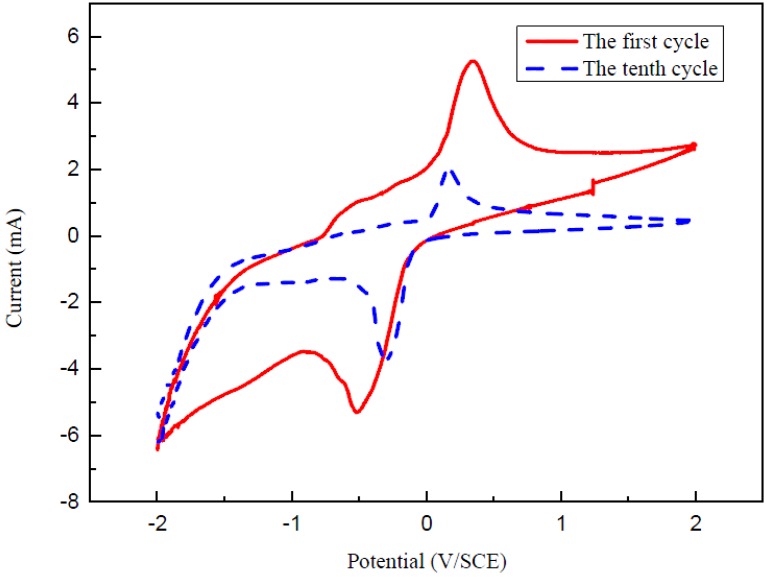
Cyclic voltammetry (CV) curve of V_2_O_5_ nanowires in 2 M KCl at 50 m·Vs^−1^ scan rate. The red curve recorded at the first cycle and the blue dash curve recorded during the 10th cycle, respectively.

For investigating the electrochemical property of V_2_O_5_ nanowires, the CV technique was used to measure the voltages of oxidation peak and reduction peak [31,32]. The typical cyclic voltammetric curve of V_2_O_5_ nanowires in 2 M KCl solution at the scan rate of 50 m·Vs^−1^ at room temperature is plotted in Figure 10. The CV was performed at a potential between −2 V and 2 V. The red CV curve recorded at the first cycle reveals that the reduction peak is located at −0.521 V and the oxidation peak is at 0.340 V, so the potential difference between these two peaks is 0.861 V. The blue dash curve recorded during the 10th cycle shows that the reduction peak is located at −0.308 V and the oxidation peak is at 0.162 V. The potential differences of the red and blue curves are 0.861 V and 0.470 V, respectively. According to these two CV curves, although the CV curve recorded during the 10th cycle becomes small compared with the red curve, the electrochemical performance and reversibility after 10 cycles still remain a good achievement, indicating that using V_2_O_5_ nanowires as a cathode is efficient in discharge-charge processes.

## 4. Conclusions

In summary, after confirmation with SEM, EDS, XRD and HRTEM analyses, the V_2_O_5_ nanowires were successfully synthesized on commercial Si substrates via the VS process without using any precursor. The mechanism of V_2_O_5_ nanowires grown by the VS process can be described as screw dislocation in the crystal plane of (110). In terms of optical and electronic properties, both examinations of photo-luminescence (PL) spectrum and current-voltage (I-V) curve were carried out. The PL excitation spectrum of V_2_O_5_ nanowires exhibits peaks at 400, 560 and 710 nm, which are caused by the various inter-band transitions from V 3d conduction band to O 2p valence band. The electrical resistivity could be assessed at 64.62 Ω·cm via I-V curve. As a result of the above analyses, the V_2_O_5_ nanowires exhibited many potential and valuable applications for optoelectronic devices. Moreover, from the CV curves recorded during the 10th cycle, the electrochemical property and durability reveal a good performance, which means that V_2_O_5_ nanowires are a promising candidate as the cathodic material in a lithium-ion secondary battery.
